# Waist Circumference as an Independent Marker of Insulin Resistance: Evidence from a Nationwide Korean Population Study

**DOI:** 10.3390/jcm14227957

**Published:** 2025-11-10

**Authors:** Sung Ha Lim, Taesic Lee, Jiyeon Oh, Kyu-Hee Hwang, Eung Ho Choi, Seung-Kuy Cha

**Affiliations:** 1 Department of Dermatology, Yonsei University Wonju College of Medicine, Wonju 26426, Republic of Korea; limsh78@yonsei.ac.kr; 2 Department of Physiology, Yonsei University Wonju College of Medicine, Wonju 26426, Republic of Korea; 3 Organelle Medicine Research Center, Yonsei University Wonju College of Medicine, Wonju 26426, Republic of Korea; ddasic123@yonsei.ac.kr; 4 Division of Data Mining and Computational Biology, Department of Convergence Medicine, Yonsei University Wonju College of Medicine, Wonju 26426, Republic of Korea; 5 Department of Global Medical Science, Yonsei University Wonju College of Medicine, Wonju 26426, Republic of Korea; 6 Institute of Mitochondrial Medicine, Yonsei University Wonju College of Medicine, Wonju 26426, Republic of Korea

**Keywords:** metabolic syndrome, obesity, type 2 diabetes, HOMA-IR, HOMA-β

## Abstract

**Background**: Waist circumference (WC) has become an essential diagnostic marker of metabolic syndrome; however, its accuracy in reflecting insulin resistance remains uncertain, particularly among Asian people who develop metabolic complications at lower levels of adiposity. **Methods**: We analyzed 20,202 adults (8886 men and 11,316 women) from the Korean National Health and Nutrition Examination Survey (KNHANES) 2015 and 2019–2021. All analyses accounted for the survey sampling weights and the complex survey design. WC, insulin resistance, and β-cell function were assessed using fasting insulin level, HOMA-IR, and HOMA-β. Associations between WC and insulin indices were evaluated using correlation analyses, stratification by WC deciles, and multivariable regression models adjusted for demographic and metabolic covariates. **Results**: WC was positively associated with HOMA-IR in both sexes (all *p* < 0.001). In the fully adjusted model, HOMA-IR remained significantly associated with WC [men, β = 0.014 (95% CI, 0.011–0.016); women, β = 0.009 (95% CI, 0.008–0.011)]. Subgroup analyses stratified by age revealed significant associations across all age groups. The association was stronger in older adults, particularly among men, whereas women exhibited significant associations across all ages with a larger age-related increase. **Conclusions**: WC is independently associated with insulin resistance in Korean adults, with stronger associations in older adults and consistent associations in women. These findings support WC as a simple yet pathophysiologically meaningful marker of metabolic risk and highlight the necessity of refining WC cutoff values for clinical screening of Asian populations and an age-specific approach for diagnosing metabolic syndrome.

## 1. Introduction

Metabolic syndrome (MetS) comprises a cluster of metabolic abnormalities that substantially increase the risk of type 2 diabetes, cardiovascular disease, and all-cause mortality [1,2]. Its prevalence continues to rise worldwide in parallel with obesity and sedentary lifestyles, posing a major public health challenge [3]. Individuals with MetS typically exhibit abdominal obesity, dyslipidemia, impaired glucose metabolism, and hypertension, and the coexistence of these risk factors accelerates atherosclerotic progression and worsens long-term outcomes [4,5]. Owing to its clinical and epidemiological significance, considerable efforts have been devoted to establishing consistent diagnostic criteria that are both pathophysiologically relevant and applicable to large-scale populations [4].

In the early conceptualization of MetS, insulin resistance was regarded as its core pathophysiological component, and the syndrome was defined by direct measurements of serum insulin [6] or derived indices such as the Homeostasis Model Assessment of Insulin Resistance (HOMA-IR) [7]. However, over time, the diagnostic criteria shifted from biochemical to anthropometric markers. In Europe and the United States (U.S.), waist circumference (WC) has replaced serum insulin as a practical indicator of central obesity [8]. Notably, the European criteria required abdominal obesity as an essential feature, whereas the U.S. guidelines treated all five diagnostic factors equally and defined MetS by the presence of any three or more. The latter diagnostic criteria have ultimately gained international consensus and formed the basis of the current definition of MetS [4,9].

Despite this historical transition, only a few studies have examined the direct relationship between circulating insulin levels and WC [8,10]. The accumulation of visceral fat contributes to insulin resistance through multiple interrelated mechanisms. Visceral adiposity is characterized by the dysregulated secretion of adipokines, including decreased adiponectin and increased leptin and resistin levels, which impair insulin receptor signaling and glucose uptake [11,12]. In addition, excess visceral fat promotes lipolysis and the release of free fatty acids into portal circulation, leading to hepatic and skeletal muscle insulin resistance [13]. Ectopic fat deposition in the liver and muscle further aggravates these effects by disrupting insulin signaling pathways such as IRS-1/PI3K/Akt [14,15]. Such mechanisms provide a physiological rationale for the close association between central obesity and insulin resistance.

Although WC is now widely accepted as a surrogate for central adiposity and metabolic risk [16,17], the extent to which WC truly reflects underlying insulin resistance, as measured by serum insulin or HOMA-IR, remains unknown. Clarifying this relationship could bridge the conceptual gap between the original insulin-based definition of MetS and its current pragmatic diagnostic criteria. Moreover, ethnic differences in body composition and metabolic susceptibility further highlight the need for population-specific evidence. Recent studies in Asian populations have demonstrated that even individuals with intermediate cardiometabolic risk exhibit a marked increase in insulin resistance and related metabolic burden, emphasizing the need for early intervention [18,19]. In addition, Asian populations have been reported to develop insulin resistance and type 2 diabetes at lower levels of obesity than Caucasian populations [20,21,22,23], implying that the WC–insulin relationship may not be identical across ethnic groups.

Therefore, we hypothesized that WC would be independently associated with insulin resistance after adjusting for potential metabolic confounders. In this study, we investigated the relationship between serum insulin indices, including fasting insulin, HOMA-IR, and WC, in the Korean National Health and Nutrition Examination Survey (KNHANES) cohort, with additional subgroup analyses by age and sex to assess potential effect modification. By simultaneously evaluating both sexes and multiple age strata within a nationally representative Asian population, our study provides a comprehensive view that has rarely been explored in previous studies. Moreover, as most prior evidence originates from Western populations, our findings add valuable population-specific evidence clarifying whether the WC criterion appropriately reflects insulin resistance in Asian people who tend to develop metabolic abnormalities at lower degrees of central obesity. Thus, our study sought to provide empirical evidence supporting the current diagnostic approach to MetS and to refine the understanding of how abdominal obesity represents metabolic risk in an Asian population.

## 2. Materials and Methods

### 2.1. Study Population

This study was based on data obtained from KNHANES, a nationwide cross-sectional survey conducted by the Korea Disease Control and Prevention Agency (KDCA, Osong, Republic of Korea). KNHANES employs a stratified, multistage probability sampling design to obtain a representative sample of the non-institutionalized Korean population. The survey consists of a health interview, physical examination, and nutrition survey and has been conducted periodically since 1998.

For the present analysis, we included participants from the KNHANES cycles conducted in 2015, 2019–2021, during which fasting serum insulin levels were measured. Intermediate cycles (2016–2018) were excluded because fasting insulin data were not collected during the survey period. The measurement procedures and laboratory protocols were standardized by the KDCA and remained consistent across the included cycles. Among the participants initially enrolled, individuals were excluded if they (1) were younger than 20 years of age and (2) had missing data on WC, fasting glucose, serum insulin, hypertension, diabetes, or dyslipidemia. After applying these criteria, 20,202 participants (8886 men and 11,316 women) were included in the final analysis.

All participants provided written informed consent, and the KNHANES protocol was approved by the Institutional Review Board of the KDCA (IRB approval was waived, IRB No. CR325341). This study was conducted in accordance with the ethical principles of the Declaration of Helsinki.

### 2.2. Waist Circumference (WC)

Information on WC was obtained from the KNHANES database, where measurements were performed by trained staff according to a standardized protocol. WC was measured to the nearest 0.1 cm at the midpoint between the lower rib margin and the iliac crest, with participants standing after normal expiration. For the present analysis, we defined abdominal obesity using sex-specific cutoff values recommended for the Korean population: ≥90 cm for men and ≥85 cm for women [24,25].

### 2.3. Serum Insulin, HOMA-IR, and HOMA-β

Data on fasting serum insulin were obtained from the KNHANES database, in which blood samples were collected after at least 8 h of overnight fasting and analyzed at a central certified laboratory using standardized immunoassay procedures.

Insulin resistance was estimated using the HOMA-IR, which was calculated as follows:

HOMA-IR = fasting insulin (µU/mL) × fasting glucose (mmol/L)/22.5

In this study, HOMA-IR was treated as a continuous variable, and participants were stratified by tertiles or deciles of HOMA-IR to explore graded associations with WC.

β-cell function was estimated using the Homeostasis Model Assessment of β-cell function (HOMA-β). This index was calculated from fasting serum insulin and glucose values obtained from the KNHANES database, according to the following formula:

HOMA-β = [20 × fasting insulin (µU/mL)]/[fasting glucose (mmol/L) − 3.5]

Higher HOMA-β values reflect greater insulin secretory activity of pancreatic β-cells. HOMA-β was analyzed as a continuous variable, and participants were also categorized by deciles of WC to assess its relationship with β-cell function.

### 2.4. Covariates

Demographic, clinical, and biochemical variables were obtained from the KNHANES database. Age and sex were recorded through standardized health interviews. Anthropometric measurements, including height, weight, and blood pressure (BP), were collected by trained examiners following standardized protocols. Body mass index (BMI) was calculated as weight (kg) divided by height squared (m^2^).

Biochemical variables were measured in fasting blood samples. Fasting glucose, total cholesterol, triglycerides (TG), high-density lipoprotein cholesterol (HDL-C), and low-density lipoprotein cholesterol (LDL-C) levels were assayed enzymatically in a central certified laboratory. Non-HDL cholesterol was calculated as the total cholesterol minus HDL-C.

For multivariable models, covariates included age, non-HDL cholesterol, and triglycerides, which were selected a priori based on their established relationships with both WC and insulin resistance.

### 2.5. Statistical Analysis

Baseline demographic, clinical, and biochemical characteristics were compared across tertiles of HOMA-IR using analysis of variance (ANOVA) for continuous variables and chi-square tests for categorical variables. Data are presented as means ± standard deviations for continuous variables and as percentages for categorical variables. The associations between WC and insulin indices (HOMA-IR and HOMA-β) were examined using Pearson’s correlation coefficients. To further evaluate dose–response relationships, participants were stratified into deciles of WC, and linear trends were assessed. Multivariable linear regression models were constructed to assess the independent associations of WC with HOMA-IR and HOMA-β. As fasting insulin and HOMA-IR exhibited right-skewed distributions, log-transformed values were used to satisfy normality assumptions. Multicollinearity between WC and BMI was examined using variance inflation factors (all VIFs < 8), which indicated acceptable levels of collinearity (Tables S1 and S2). Regression coefficients (β) and 95% confidence intervals (CIs) were reported separately for men and women. Model fit was assessed using R^2^ values and residual analyses to confirm the normality and homoscedasticity of the residuals.

All analyses accounted for the complex, stratified, and multistage sampling design of the KNHANES by applying survey weights in accordance with official analytic guidelines. Statistical analyses were performed using the R software (version 4.2.2; R Foundation for Statistical Computing, Vienna, Austria). Statistical significance was defined as a two-sided *p*-value of <0.05.

## 3. Results

### 3.1. Study Population and Baseline Characteristics

To address the relationship between abdominal obesity and serum insulin levels, we utilized data from the KNHANES, which comprised 8886 men and 11,316 women. The KNHANES predominantly included a middle-aged population, with mean ages of 47.9 years in men and 49.8 years in women. WC approximated a Gaussian distribution in both sexes, with a mean of 86.4 cm and 79.9 cm in men and women, respectively.

Table 1 and Table 2 summarizes the demographic, clinical, and biochemical characteristics of participants according to the recommended cutoff of WC in Asia and the WHO [25,26]. For men, the well-known cutoffs of 90 and 102 cm of WC were applied, whereas the WC categorized distribution of women proportions would differ markedly from those of men; therefore, values of 85 and 95 cm were chosen. Across increasing WC categories, both men and women exhibited progressively higher age, BP, and prevalence of antihypertensive medication (AHM) use, diabetes, and lipid-lowering drug (LLD) use. In parallel, non-HDL cholesterol, TG, insulin levels, HOMA-IR, and HOMA-β increased, whereas HDL-C levels declined. These findings indicate that greater abdominal adiposity is accompanied by a more adverse cardiometabolic profile in both the sexes.

### 3.2. Association of WC with Insulin Resistance and Beta-Cell Function

**Figure 1:**
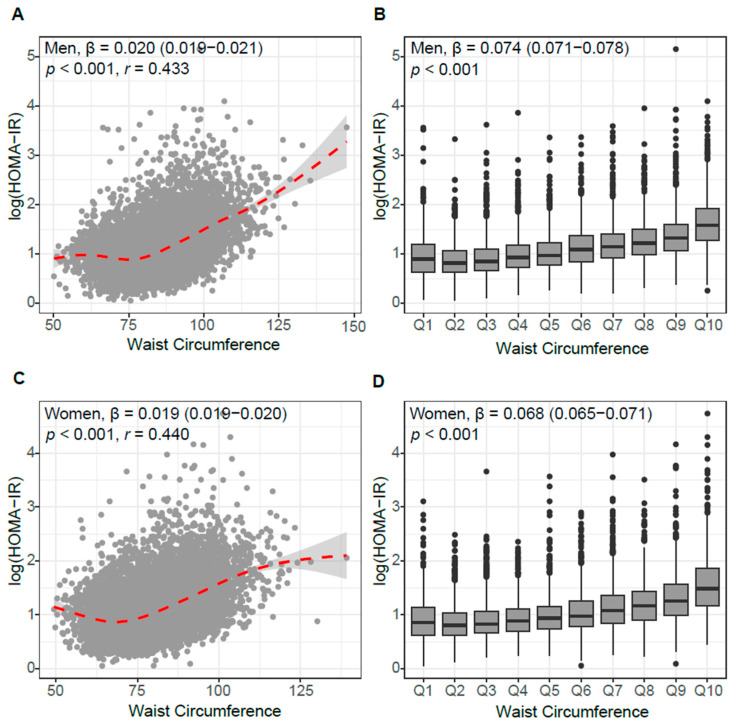
Association between waist circumference (WC) and insulin resistance (HOMA-IR). (**A**,**C**) Scatter plots with a locally weighted regression curve (red dashed line with 95% confidence in-terval) in men (**A**) and women (**C**). Box plots of HOMA-IR across WC deciles in men (**B**) and women (**D**). Each box represents the interquartile range (IQR) with the median line, and whiskers indicate 1.5 × IQR. Outliers are plotted as individual points.

Pearson’s correlation analysis revealed that the continuous distribution of WC correlated well with HOMA-IR in men (β = 0.020, *p* < 0.001) (Figure 1A). Their positive linear relationships remained after transforming continuous WC into deciles (β = 0.074, *p* < 0.001) (Figure 1B). In women, the correlation between WC and HOMA-IR was significant, irrespective of the type of WC distribution (Figure 1C; β = 0.019, *p* < 0.001; and Figure 1D; β = 0.068, *p* < 0.001). Comparing men and women, the difference in the association between WC and IR was minor.

**Figure 2:**
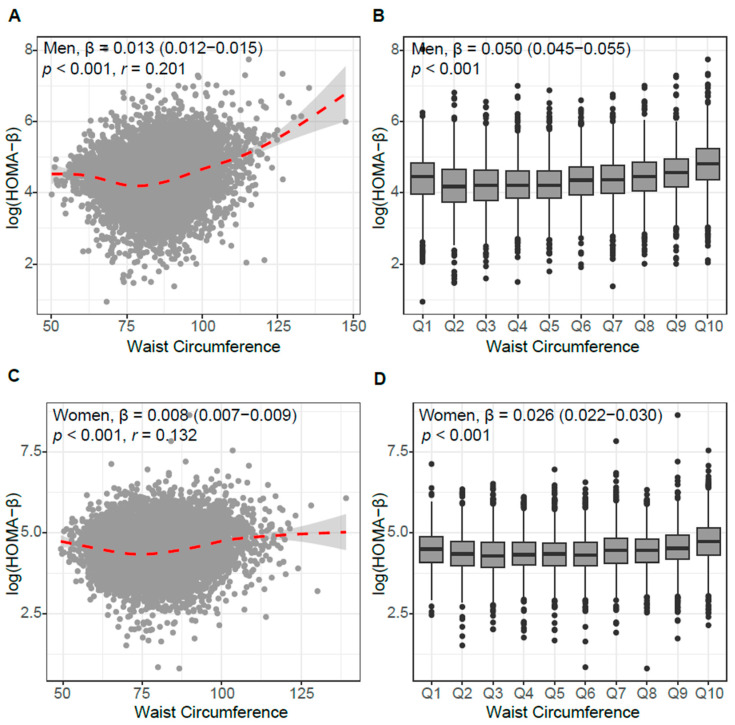
Association between waist circumference (WC) and β-cell function (HOMA-β). (**A**,**C**) Scatter plots with locally weighted regression curves (red dashed lines with 95% confidence in-terval) in men (**A**) and women (**C**). Box plots of HOMA-β across deciles of WC deciles in men (**B**) and women (**D**). Each box represents the interquartile range (IQR) with the median line, and whiskers indicate 1.5 × IQR. Outliers are plotted as individual points.

WC also correlated with pancreatic β-cell function, as assessed by HOMA-β (Figure 2).However, the strength of the association between WC and HOMA-IR was greater than that between WC and HOMA-β.

### 3.3. Independent Association of WC and HOMA-IR

To examine whether there was an independent association between WC and serum insulin level, we employed a multivariable model. Note that all multivariable analyses were conducted using weighted population data. First, in men, a multivariable linear regression model including age (Model 1, Table 3) revealed that WC was significantly associated with HOMA-IR. Model 2, which included age, AHM, diabetes, and LLD, identified an independent association between WC and HOMA-IR in men. After further adjustment for BMI as well as behavioral covariates including smoking status, alcohol consumption, and physical activity in Model 3, the β estimates were attenuated compared with Model 2, but the association between WC and HOMA-IR remained statistically significant, indicating that WC provides information beyond general adiposity. The final complex multivariable model also revealed that WC was significantly independent association with HOMA-IR (Model 4). Similarly, in women subgroup, a robust association between WC and HOMA-IR was observed, regardless of the model type (Table 3).

### 3.4. Linearity Between WC and Insulin Resistance

**Figure 3:**
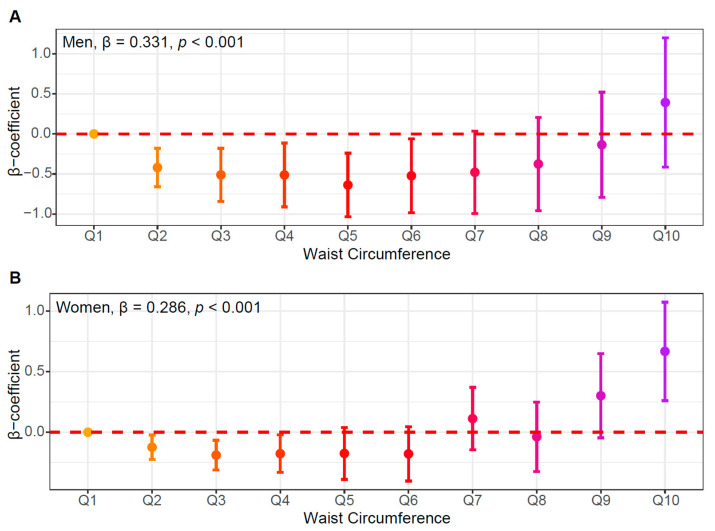
Multivariable-adjusted associations between waist circumference (WC) and insulin resistance (HOMA-IR). (**A**) Men and (**B**) women. β-coefficients (dots) and 95% confidence intervals (bars) are plotted for each WC quantile (Q1–Q10), estimated from multivariable linear regression models adjusted for age, lifestyle factors, and comorbidities.

To further interrogate the dose–response relationship between WC and insulin resistance, we divided WC into 10 quantiles (deciles) and examined its association with HOMA-IR using multivariable-adjusted models. In both men and women, WC showed a clear positive linear association with HOMA-IR across quantiles (Figure 3A,B; men, β = 0.331, *p* < 0.001; women, β = 0.286, *p* < 0.001). The β coefficients progressively increased in higher WC quantiles, indicating a stronger association in individuals with greater abdominal adiposity.

**Figure 4:**
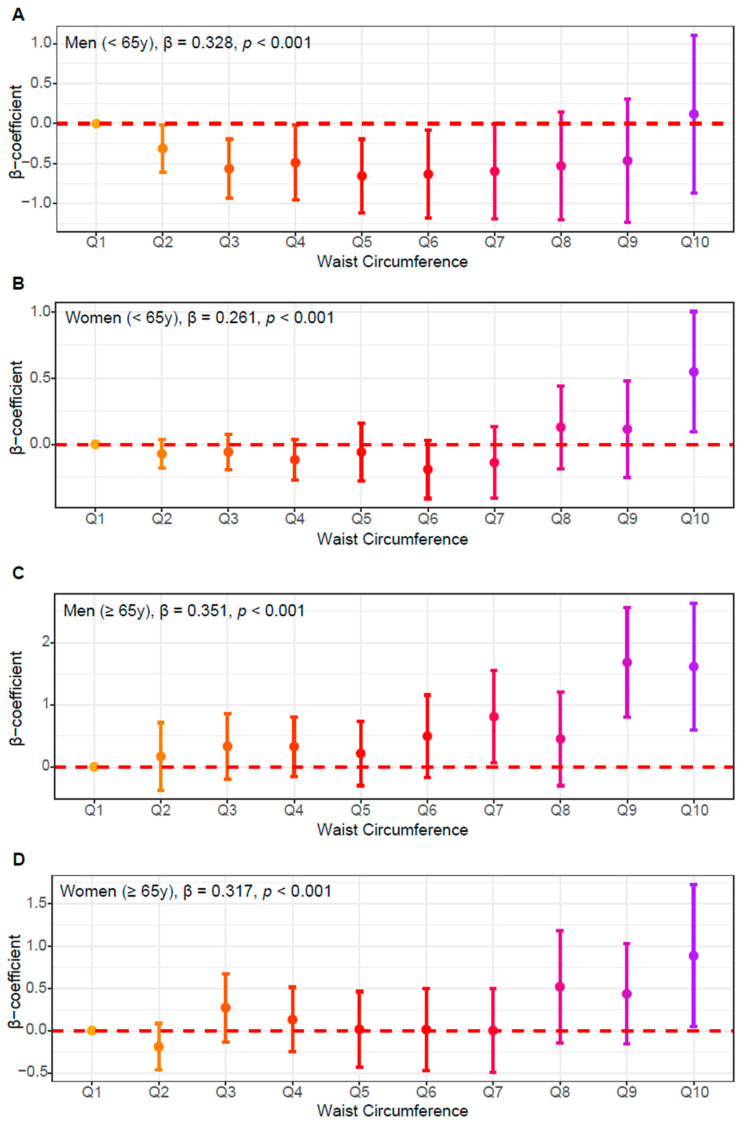
Subgroup analysis of waist circumference (WC) and insulin resistance by sex and age group. (**A**) Young and middle-aged (<65 years) men, and (**B**) women, (**C**) older (≥65 years) men, and (**D**) older women. β-coefficients (dots) and 95% confidence intervals (bars) are plotted across WC quantiles (Q1–Q10), estimated from multivariable linear regression models adjusted for age, lifestyle factors, and comorbidities.

Prior studies have suggested that age-related declines in insulin sensitivity are largely attributable to increased adiposity and alterations in mitochondrial function, rather than chronological aging itself. While men generally exhibit higher mitochondrial ATP production, women tend to maintain greater insulin sensitivity [27]. Therefore, we performed a stratified analysis by age and sex to examine how the relationship between WC and insulin resistance differed across these groups (Figure 4). Among individuals younger than 65 years, both men and women exhibited significant positive associations between WC and HOMA-IR (men, β = 0.328, *p* < 0.001; women, β = 0.261, *p* < 0.001). In older adults (≥65 years), this association persisted and appeared slightly stronger (men, β = 0.351, *p* < 0.001; women, β = 0.317, *p* < 0.001), suggesting that the metabolic burden of central obesity may accumulate with age. Detailed sample sizes and mean WC values for each decile and age-sex subgroup are provided in Tables S3 and S4.

## 4. Discussion

In this nationwide study of Korean adults, we found that WC was strongly and independently associated with insulin resistance, as assessed by HOMA-IR, in both men and women. Although the standardized β-coefficients of WC were relatively small, these findings were consistent and robust across the models. The modest individual-level effects may still be clinically relevant at the population level, as small shifts in insulin resistance can cumulatively contribute to a substantial metabolic risk in the general population. This relationship was further supported by consistent findings across the correlation, decile stratification, and multivariable regression analyses, highlighting the robustness of WC as a surrogate marker of insulin resistance. The dose–response pattern was also notable; while WC was only modestly related to insulin resistance at lower quantiles, the slope steepened substantially at the highest WC categories, indicating a potential threshold effect beyond which the metabolic burden of abdominal obesity is markedly amplified. Subgroup analyses further revealed that this association was more evident in older adults, suggesting that the adverse effects of central adiposity accumulate with age.

Previous studies in Western populations have reported similar associations between central adiposity and insulin resistance [8,17]; however, large-scale evidence from Asian cohorts has been limited. Using nationally representative Korean health data, our findings extend prior observations by demonstrating that WC robustly captures biochemical insulin resistance even in a population with lower mean adiposity. Given that Asian people tend to develop metabolic complications at lower BMI and WC values than Western counterparts, these results provide population-specific evidence reinforcing WC as a pathophysiologic al crucial indicator of metabolic risk. Furthermore, by incorporating sex- and age-stratified analyses, the study provides novel insight into demographic heterogeneity in the WC–insulin relationship and showed the importance of population-tailored interpretation of WC in both clinical and epidemiological contexts.

The strong association between WC and HOMA-IR aligns with the central role of visceral adiposity in the pathogenesis of insulin resistance. Excess abdominal fat is metabolically active and secretes bioactive mediators such as free fatty acids, adipokines, and proinflammatory cytokines, which interfere with insulin signaling in muscle and liver [16,28,29,30]. These alterations reduce peripheral glucose uptake and enhance hepatic gluconeogenesis, thereby increasing insulin requirements [31]. The non-linear pattern observed in our analysis suggests that once visceral fat accumulation exceeds a critical threshold, compensatory mechanisms fail and insulin resistance escalates disproportionately [32,33].

We also elucidated the relationship between WC and HOMA-β to explore the pancreatic responses accompanying insulin resistance. The association between WC and HOMA-β likely reflects compensatory β-cell hypersecretion in response to increasing insulin resistance. As peripheral tissues become less responsive to insulin, pancreatic β-cells increase their insulin output to maintain euglycemia. Our finding of a modest but significant association between WC and HOMA-β in both sexes supports this compensation. Subsequently, persistent β-cell stress may lead to eventual secretory failure, contributing to the transition from insulin resistance to overt type 2 diabetes.

Subgroup analyses provided further insights into age-dependent differences. Previous studies have reported that age-related decline in insulin sensitivity is primarily mediated by increased adiposity rather than aging itself [27]. Based on these findings, we performed age-stratified analyses to examine whether aging per se modifies the relationship between WC and insulin resistance. Interestingly, our data revealed a stronger association between WC and HOMA-IR in older adults, suggesting that aging may potentiate the metabolic impact of abdominal adiposity. This finding implies that aging not only reflects accumulated adiposity but also interacts with it through tissue-level remodeling and mitochondrial dysfunction. Aging skeletal muscles exhibit mitochondrial dysfunction, intramyocellular lipid deposition, oxidative stress, and inflammatory signaling, all of which impair insulin signaling and contribute to insulin resistance [34,35]. In parallel, aging adipose tissue undergoes remodeling with increased visceral adiposity, adipocyte dysfunction, and a heightened inflammatory milieu, further aggravating metabolic dysregulation [36]. A cross-sectional study in older adults also reported that lower appendicular lean mass and higher abdominal fat mass were independently associated with elevated HOMA-IR [37]. Clinically, these findings suggest that the same degree of WC confers a greater metabolic risk in older individuals than in younger individuals, implying the need for age-conscious interpretation of anthropometric risk markers.

Furthermore, sex-specific differences were also evident. In both men and women, the positive association between WC and HOMA-IR remained statistically significant across all age groups. The absolute strength of association was greater in men, particularly in older adults, indicating that aging amplifies the metabolic burden of abdominal obesity in males. However, the increase in β with age was proportionally larger in women, suggesting that the age-related rise in insulin resistance associated with central adiposity may be more pronounced in females. While diagnostic criteria already account for sex-specific WC thresholds (≥85 cm for women and ≥90 cm for men), our findings suggest that women may nonetheless be more vulnerable to the metabolic consequences of visceral adiposity. This is in line with prior studies reporting that women may be more metabolically susceptible to central fat accumulation at relatively lower WC levels, potentially due to differences in fat distribution, sex hormones, or adipokine secretion [38,39,40]. In particular, estrogen decline after menopause may accelerate visceral fat accumulation and impair mitochondrial function, thereby exacerbating insulin resistance, whereas lower lean body mass in women compared with men may further contribute to these sex-specific metabolic differences [41].

Our results also harbor implications for international diagnostic criteria for MetS. Current WC thresholds are largely derived from Western populations, where higher levels of obesity are common. However, Asian people are known to develop insulin resistance and type 2 diabetes at lower levels of adiposity [21]. The sharp rise in HOMA-IR at comparatively modest WC levels in our study reinforces the notion that lower WC cutoffs may be more appropriate for Asian populations. Therefore, the observed pathophysiological link between WC and insulin resistance in this cohort suggests the need for population- and age-specific refinement of the diagnostic criteria. Incorporating ethnic-based WC thresholds into clinical and screening practices may improve the early detection and prevention of metabolic disorders in Asian populations.

This study has several limitations that merit consideration. First, the cross-sectional design precludes causal inference between WC and insulin resistance. However, previous population-based studies have demonstrated that greater abdominal adiposity is strongly associated with and predictive of insulin resistance and type 2 diabetes, supporting the biological plausibility of our findings [20,37]. Second, fasting insulin and HOMA-derived indices, which are widely accepted, are indirect measures of insulin resistance and β-cell function; gold-standard techniques, such as the euglycemic–hyperinsulinemic clamp [42], were not available in our database. Third, residual confounding factors from unmeasured variables, including dietary intake, socioeconomic status, and genetic variation, cannot be excluded. Finally, our findings pertain to the Korean population and may not be directly generalizable to other ethnic groups, although they are likely relevant to other Asian populations with similar body composition profiles.

## 5. Conclusions

In summary, WC represents a simple yet physiologically meaningful marker that reflects insulin resistance in Korean adults, whereas its association with β-cell function appears relatively modest. Given its feasibility in both clinical practice and population-based surveys, WC represents a practical marker for early detection and risk stratification of metabolic disorders. Moreover, integrating WC with non-invasive insulin resistance indices such as the triglyceride–glucose (TyG) index or the metabolic score for insulin resistance (METS-IR) may further enhance its clinical utility as a screening tool, particularly in Asian populations where these indices are increasingly adopted. Our findings further highlight the need to refine WC cutoff values not only for Asian populations, who develop metabolic risk at lower levels of adiposity, but also with consideration of age and sex, as the metabolic burden of abdominal obesity appears to intensify in older adults and differ between men and women.

## Figures and Tables

**Table 1:** Demographic characteristics of the study participants according to waist circumference (WC) categories.

	Men					Women				
WC, cm (*n*)	Total (8886)	<90 (5662)	90–102 (2689)	>102 (535)	*p* for Trend	Total (11,316)	<85 (7865)	85–95 (2511)	>95 (940)	*p* for Trend
Age, y	47.9 ± 20.40	45 ± 21.26	53.9 ± 17.31	48.9 ± 18.86	<0.001	49.8 ± 19.03	45.6 ± 18.85	59.8 ± 15.20	59.3 ± 16.34	<0.001
BMI, kg/m^2^	24.3 ± 3.63	22.4 ± 2.54	26.7 ± 2.04	31.6 ± 3.34	<0.001	23.4 ± 3.77	21.7 ± 2.45	26.1 ± 2.14	30.6 ± 3.54	<0.001
WC, cm	86.4 ± 10.52	80.4 ± 7.37	94.8 ± 3.28	107.6 ± 5.69	<0.001	79.9 ± 10.53	74.5 ± 6.67	89.2 ± 2.74	100.7 ± 5.57	<0.001
SBP, mmHg	120.4 ± 14.85	118 ± 14.83	124.5 ± 14.1	125.7 ± 12.72	<0.001	117 ± 17.55	113.4 ± 16.55	124.4 ± 17.33	127.6 ± 15.6	<0.001
DBP, mmHg	75.7 ± 10.53	73.9 ± 10.16	78.5 ± 10.26	80.1 ± 11.40	<0.001	72.8 ± 9.51	71.7 ± 9.32	75 ± 9.41	76.7 ± 9.41	<0.001
AHM, *n*	1921 (21.6)	838 (14.8)	884 (32.9)	199 (37.2)	<0.001	2463 (21.8)	1029 (13.1)	968 (38.6)	466 (49.6)	<0.001
Diabetes, *n*	928 (10.4)	424 (7.5)	399 (14.8)	105 (19.6)	<0.001	989 (8.7)	389 (4.9)	398 (15.9)	202 (21.5)	<0.001
LLD, *n*	1019 (11.5)	463 (8.2)	446 (16.6)	110 (20.6)	<0.001	1834 (16.2)	835 (10.6)	703 (28)	296 (31.5)	<0.001
Smoking status	2413 (27.2)	1473 (26)	793 (29.5)	147 (27.5)	0.004	504 (4.5)	323 (4.1)	119 (4.7)	62 (6.6)	0.002
Alcohol consumption	1999 (22.5)	1116 (19.7)	745 (27.7)	138 (25.8)	<0.001	917 (8.1)	653 (8.3)	187 (7.4)	77 (8.2)	0.39
Physical activity	1807 (20.3)	1249 (22.1)	474 (17.6)	84 (15.7)	<0.001	1743 (15.4)	1387 (17.6)	267 (10.6)	89 (9.5)	<0.001

Summary statistics for continuous and categorical variables are presented as mean ± standard deviation or number (percentage), respectively. *p* for trend values were derived from one-way ANOVA for continuous variables and χ^2^ tests for categorical variables across WC categories. Abbreviations: WC, waist circumference; BMI, body mass index; SBP, systolic blood pressure; DBP, diastolic BP; AHM, antihypertensive medication; LLD, lipid-lowering drug.

**Table 2:** Metabolic profiles of the study participants according to waist circumference (WC) categories.

	Men					Women				
WC, cm (*n*)	Total (8886)	<90 (5662)	90–102 (2689)	>102 (535)	*p* for Trend	Total (11,316)	<85 (7865)	85–95 (2511)	>95 (940)	*p* for Trend
HDL-C, mg/dL	48.1 ± 11.43	50 ± 11.63	45.1 ± 10.33	42.8 ± 9.44	<0.001	54.8 ± 12.72	56.8 ± 12.75	50.8 ± 11.64	49.1 ± 10.87	<0.001
Non-HDL-C, mg/dL	136.2 ± 38.31	132.1 ± 37.11	143.1 ± 39.09	144.8 ± 40.46	<0.001	135.5 ± 36.74	133 ± 35.22	141.1 ± 38.59	141.2 ± 41.51	<0.001
TG, mg/dL	144.6 ± 119.41	126.1 ± 107.78	175.6 ± 131.79	183.5 ± 129.27	<0.001	111.1 ± 70.29	99.1 ± 62.62	134.4 ± 76.55	148.4 ± 83.53	<0.001
Insulin, μU/mL	10.1 ± 10.20	8.1 ± 7.01	12.3 ± 12.53	20 ± 15.87	<0.001	9.3 ± 8.43	7.8 ± 6.36	11.2 ± 8.17	16.9 ± 15.86	<0.001
Glc, mg/dL	103.3 ± 24.16	99.8 ± 20.73	108.3 ± 26.65	115.4 ± 34.27	<0.001	99.2 ± 21.65	95.3 ± 17.1	106 ± 25.87	112.8 ± 31.22	<0.001
HOMA-IR	2.7 ± 3.49	2 ± 2.09	3.4 ± 4.68	5.8 ± 5.4	<0.001	2.4 ± 2.89	1.9 ± 1.77	3.1 ± 3.08	5 ± 6.24	<0.001
HOMA-β	101.4 ± 99.73	89 ± 82.93	112.7 ± 100.24	175.8 ± 184.86	<0.001	101.8 ± 95.5	94.7 ± 73.28	108.6 ± 133.2	143 ± 123.49	<0.001

Summary statistics are presented as mean ± standard deviation. *p* for trend values were derived from one-way ANOVA across WC categories. Abbreviations: WC, waist circumference; HDL-C, high-density lipoprotein cholesterol; TG, triglyceride; Glc; fasting serum glucose; HOMA-IR, Homeostatic Model Assessment of Insulin Resistance; HOMA-β, Homeostatic Model Assessment of beta-cell function.

**Table 3:** Multivariable linear regression analyses of the association between waist circumference (WC) and insulin resistance (HOMA-IR) in Korean adults.

		Men						Women				
	β	*se*	95% CI	*t*	*p*	*R* ^2^	β	*se*	95% CI	*t*	*p*	*R* ^2^
Model 1	0.025	0.001	0.024–0.026	45.2	<0.001	0.269	0.025	0.001	0.024–0.026	47.8	<0.001	0.264
Model 2	0.024	0.001	0.023–0.025	42.9	<0.001	0.296	0.023	0.001	0.022–0.024	45.2	<0.001	0.305
Model 3	0.017	0.001	0.014–0.02	12.4	<0.001	0.320	0.015	0.001	0.013–0.017	13.8	<0.001	0.331
Model 4	0.014	0.001	0.011–0.016	11.5	<0.001	0.465	0.009	0.001	0.008–0.011	10.2	<0.001	0.497

Linear regression was performed using continuous WC and HOMA-IR as the independent and dependent variables, respectively. Model 1: adjusted by age. Model 2: adjusted by Model 1 + antihypertensive medication + diabetes + LLD. Model 3: adjusted by Model 2 + body mass index + systolic blood pressure + smoking status + alcohol consumption status + physical activity. Model 4: adjusted by Model 3 + serum glucose, HDL-C, non-HDL-C, and TG. Abbreviations: WC, waist circumference; OR, odds ratio; CI, confidence interval.

## Data Availability

All data supporting the findings of this study are included in the main text and Supplementary Information Files. Additional datasets and materials are available from corresponding authors upon request.

## Supplementary Materials

The following supporting information can be downloaded at: https://www.mdpi.com/article/10.3390/jcm14227957/s1, Table S1: Multivariable linear regression analyses were conducted to examine the association between waist circumference (WC) and log-transformed HOMA-IR in Korean men; Table S2: Multivariable linear regression analyses were conducted to examine the association between waist circumference (WC) and log-transformed HOMA-IR in Korean women; Table S3: Sample sizes and mean waist circumference (WC) values by decile group of WC among Korean adults; Table S4: Sample sizes and mean waist circumference (WC) values by decile group, stratified by age (<65 and ≥65 years) and sex in Korean adults.

## Abbreviations

The following abbreviations are used in this manuscript:

MetS	Metabolic syndrome
WC	Waist circumference
BMI	Body mass index
HDL-C	High-density lipoprotein cholesterol
LDL-C	Low-density lipoprotein cholesterol
KNHANES	Korea National Health and Nutrition Examination Survey
KDCA	Korea Disease Control and Prevention Agency
HOMA-IR	Homeostasis Model Assessment of Insulin Resistance
HOMA-β	Homeostasis Model Assessment of β-cell function
ANOVA	Analysis of variance
AHM	Antihypertensive medication
LLD	Lipid-lowering drug
