# Supplementary material for: Waist Circumference as an Independent Marker of Insulin Resistance: Evidence from a Nationwide Korean Population Study
Source: J Clin Med. 2025 Nov 10;14(22):7957. doi: 10.3390/jcm14227957 (PMC12653953; doi:10.3390/jcm14227957)
Supplement: Supplementary file 1 [file jcm-14-07957-s001.zip › jcm-3946284-supplementary.pdf]

Supplementary Materials for

Waist Circumference as an Independent Marker of Insulin Resistance: Evidence from a Nationwide Korean Population Study

Sung Ha Lim [1,2†], Taesic Lee [3,4†], Jiyeon Oh [2,3,5,6], Kyu-Hee Hwang [2,3,5,6], Eung Ho Choi [1*], and Seung-Kuy Cha [2,3,5,6,*]

[1] Department of Dermatology, Yonsei University Wonju College of Medicine, Wonju, Republic of Korea
[2] Department of Physiology, Yonsei University Wonju College of Medicine, Wonju, Republic of Korea
[3] Organelle Medicine Research Center, Yonsei University Wonju College of Medicine, Wonju, Republic of Korea
[4] Division of Data Mining and Computational Biology, Department of Convergence Medicine, Yonsei University Wonju College of Medicine, Wonju, Republic of Korea
[5] Department of Global Medical Science, Yonsei University Wonju College of Medicine, Wonju, Republic of Korea
[6] Institute of Mitochondrial Medicine, Yonsei University Wonju College of Medicine, Wonju, Republic of Korea
† These authors contributed equally

Supplementary Table S1. Multivariable linear regression analyses were conducted to examine the association between waist circumference (WC) and log-transformed HOMA-IR in Korean men. Regression coefficients (β), standard errors (SE), t-values, p-values, and variance inflation factors (VIF) are presented. VIF values were used to assess multicollinearity among covariates; all VIFs were below 10, indicating no severe collinearity.

Variable	Estimate (β)	SE	t-value	P-value	VIF
Intercept	-7.1161	0.6671	-10.6679	0.0000	NA
WC	0.0421	0.0094	4.4854	0.0000	7.5169
Age	-0.0346	0.0021	-16.3661	0.0000	1.7880
AHM	0.3154	0.0964	3.2705	0.0011	1.5307
Diabetes	-1.1425	0.2144	-5.3277	0.0000	1.5580
LLD	0.1402	0.1161	1.2080	0.2275	1.3586
BMI	0.1316	0.0312	4.2224	0.0000	6.8861
SBP	0.0041	0.0026	1.5606	0.1191	1.2675
Alcohol	-0.5106	0.0751	-6.7992	0.0000	1.1898
Exercise	-0.4085	0.0704	-5.8053	0.0000	1.0270
Smoking	-0.2494	0.0698	-3.5714	0.0004	1.0678
Glc	0.0521	0.0049	10.7150	0.0000	1.5029
HDL	-0.0121	0.0037	-3.2322	0.0013	1.3349
nonHDL	-0.0055	0.0009	-6.1963	0.0000	1.4716
TG	0.0017	0.0004	3.9277	0.0001	1.5405

Supplementary Table S2. Multivariable linear regression analyses were conducted to examine the association between waist circumference (WC) and log-transformed HOMA-IR in Korean women. Regression coefficients (β), standard errors (SE), t-values, p-values, and variance inflation factors (VIF) are presented. VIF values for WC and BMI were approximately 7 and 6, respectively, indicating no significant multicollinearity.

Variable	Estimate (β)	SE	t-value	P-value	VIF
Intercept	-5.6191	0.4662	-12.0517	0.0000	NA
WC	0.0251	0.0058	4.3018	0.0000	7.3010
Age	-0.0387	0.0020	-19.0758	0.0000	2.1775
AHM	0.4233	0.0826	5.1265	0.0000	1.5531
Diabetes	-0.7267	0.1763	-4.1212	0.0000	1.5485
LLD	0.1829	0.0984	1.8592	0.0634	1.4492
BMI	0.1154	0.0154	7.5177	0.0000	6.0347
SBP	0.0012	0.0015	0.7908	0.4294	1.5285
Alcohol	-0.4498	0.0823	-5.4626	0.0000	1.1221
Exercise	-0.2760	0.0477	-5.7916	0.0000	1.0288
Smoking	0.0926	0.1339	0.6916	0.4894	1.0616
Glc	0.0563	0.0046	12.3223	0.0000	1.5477
HDL	-0.0106	0.0017	-6.1248	0.0000	1.3386
nonHDL	-0.0033	0.0008	-4.1829	0.0000	1.4744
TG	0.0048	0.0008	5.9912	0.0000	1.5530

Supplementary Table S3. Sample sizes and mean waist circumference (WC) values by decile group of WC among Korean adults. Values represent the mean WC (cm) and corresponding sample sizes (N) for each decile group used in the correlation and regression analyses of HOMA-IR and HOMA-β.

	Men		Women	
**Decile**	**Mean WC**	**N**	**Mean WC**	**N**
1	67.307	907	63.229	1154
2	75.673	879	68.887	1125
3	79.948	892	72.367	1141
4	83.006	892	75.287	1133
5	85.644	889	77.984	1110
6	88.056	894	80.715	1168
7	90.421	893	83.760	1129
8	93.158	880	86.931	1103
9	96.751	873	90.950	1128
10	104.784	887	99.632	1125

Supplementary Table S4. Sample sizes and mean waist circumference (WC) values by decile group, stratified by age (<65 and ≥65 years) and sex in Korean adults. Values represent the mean WC (cm) and corresponding sample sizes (N) for each decile group, stratified by sex and age (<65 years and ≥65 years).

	Men				Women			
	< 65 y		≥ 65 y		< 65 y		≥ 65 y	
**Decile**	**Mean**	**N**	**Mean**	**N**	**Mean**	**N**	**Mean**	**N**
1	65.896	664	72.643	234	62.263	843	70.183	304
2	74.494	674	79.557	228	67.464	835	76.437	286
3	78.930	654	83.274	224	70.634	853	79.468	291
4	81.964	655	85.875	221	73.305	857	82.129	304
5	84.637	665	88.132	241	75.787	827	84.599	289
6	87.157	661	90.100	214	78.316	839	86.676	291
7	89.631	664	92.135	234	81.075	837	89.060	301
8	92.471	661	94.517	221	84.319	818	91.517	287
9	96.273	660	97.831	230	88.466	843	94.889	290
10	104.955	660	104.331	221	98.031	828	102.033	293
